# Supplementary material for: Stimulating Mitochondrial Biogenesis with Deoxyribonucleosides Increases Functional Capacity in ECHS1-Deficient Cells
Source: Int J Mol Sci. 2022 Oct 20;23(20):12610. doi: 10.3390/ijms232012610 (PMC9604038; doi:10.3390/ijms232012610)
Supplement: Supplementary file 1 [file ijms-23-12610-s001.zip › Figure S1.pdf]

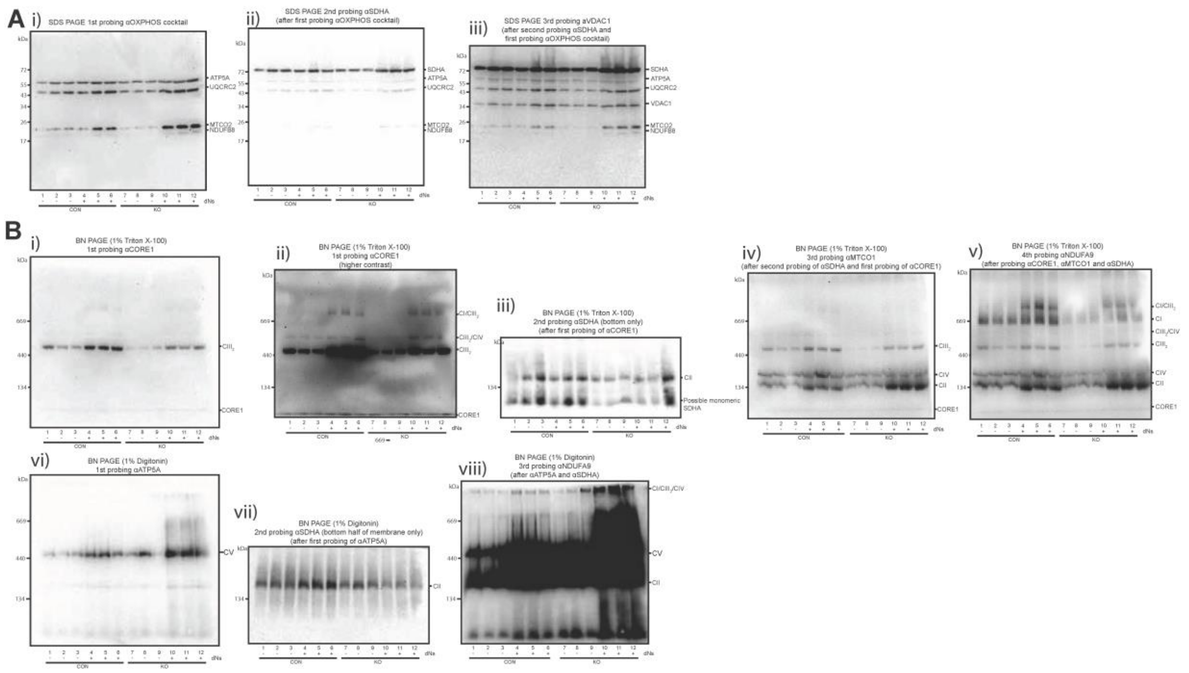

Figure S1. (A) SDS PAGE was transferred to PVDF membrane via Western blot before being probed with antibodies against OXPHOS cocktail (i), SDHA (ii) and VDAC1 (iii). (**B)** BN PAGE (1% Triton X-100 solubilised samples) was transferred to PVFD membrane via Western blot and probed with antibodies against CORE1 (CIII2, CIII2/CIV, i and ii), SDHA (CII, iii), MTCO1 (CIV, iv) and NDFUA9 (CI, v). BN PAGE (1% Digitonin solubilised samples) was transferred to PVDF membrane via Western blot and probed with antibodies against ATP5A (CV, vi), SDHA (CII, vii) and NDUFA9 (CI/CIII2/CIV, viii). Images shown are representative of three independent experiments.
